# Family health as a mediator between work–family conflict and depression in working parents: a cross-sectional study

**DOI:** 10.3389/fpsyg.2026.1808855

**Published:** 2026-05-26

**Authors:** Min Yang, Chaochan Su, Meng Yang, Qiuxia Huang

**Affiliations:** 1 Guangdong Province Hospital for Occupational Disease Prevention and Treatment, Guangzhou, China; 2 The Second Affiliated Hospital, Guangzhou Medical University, Guangzhou, China; 3 Guangzhou Nansha District Center for Disease Control and Prevention, Guangzhou, China

**Keywords:** depression, family health, mediation analysis, work–family conflict, workplace

## Abstract

**Background:**

Although there is a growing body of research on work–family conflict and its impact on mental health, significant gaps in the literature remain.

**Objective:**

To investigate the mediating role of family health in the association between WFC and depression among Chinese working parents.

**Methods:**

A cross-sectional study was conducted using data from the 2021 Psychology and Behavior Investigation of Chinese Residents, which included 5,068 working parents. Work–family conflict was measured using the Work–Family Conflict Scale (WAFCS), family health was assessed using the Short Form of the Family Health Scale (FHS-SF), and depression was evaluated using the Patient Health Questionnaire-9 (PHQ-9). Logistic regression models and mediation analysis were employed to examine the relationships between WFC, family health, and depression.

**Results:**

The study found that 16.0% of participants reported symptoms of depression. Higher levels of WFC were significantly associated with an increased risk of depression (OR = 1.01, 95% CI: 1.01–1.02), while higher family health scores were associated with a reduced risk of depression (OR = 0.95, 95% CI: 0.93–0.97). Family health partially mediated the relationship between WFC and depression, accounting for 14.27% of the total effect. Younger parents (under 35 years of age) and those with multiple illnesses were at higher risk of depression.

**Conclusion:**

Family health plays a mediating role in the relationship between WFC and depression among Chinese working parents. Although the mediation effect is modest, it represents a large-scale investigation in a Chinese-speaking population, providing actionable metrics for public health screening. This study makes three contributions. Theoretically, it integrates COR theory and the W-HR model by positioning family health as a dynamic mediating resource; empirically, it provides a first large-scale, nationally representative quantification of this indirect pathway among working parents; and practically, it identifies a concrete, modifiable intervention target with actionable metrics for public health screening. These findings highlight the importance of holistic approaches that address both individual- and family-level factors in mental health interventions.

## Introduction

### Background

The intersection of work and family life represents a critical challenge in modern society (Cavagnis et al., 2023), particularly as the boundaries between these two domains become increasingly blurred. Work and family are the two primary aspects of life for most working adults, and the interaction between these domains can often be problematic, leading to what is known as work–family conflict (WFC) (Grzywacz and Marks, 2000). The complexity of WFC has been exacerbated by significant social trends, including greater participation of women in the workforce (Rajabi-Gilan et al., 2025; Wandschneider et al., 2022), the rise of single-parent and dual-earner families, and growing eldercare responsibilities. Individuals are routinely required to navigate competing role expectations across work and family domains. In fast–growing economic regions, such as China, these strains are further intensified by rapid urbanization, evolving childcare demands, growing eldercare responsibilities, and the cultural emphasis on multi-generational family interdependence. Consequently, WFC has emerged as a pervasive psychosocial risk with profound implications for the working force’s mental health.

Depression represents one of the most detrimental outcomes of chronic WFC. Depression symptoms, as a typical negative emotional state, are prevalent in various aspects of life (Li et al., 2024) and are manifested by symptoms such as sleep disturbances, fatigue, lack of concentration, loss of appetite, and lack of interest in daily experiences, leading to impaired interpersonal, social, and occupational functioning (Zhou et al., 2018). Psychologically, WFC is linked to depression, anxiety disorders, emotional exhaustion, and burnout (Liu and Ci, 2025). These outcomes can create a negative cycle, in which one consequence often leads to or exacerbates others, affecting both the quality of work life and non-work life (Cha and Lee, 2024).

### Problem statement

Although extensive research has documented a robust direct association between WFC and depression, the precise pathways through which WFC leads to depressive symptomatology remain incompletely understood. Existing studies have largely focused on establishing the magnitude of the direct effect, while little attention has been paid to the mediating mechanisms that transmit WFC into individual psychopathology.

Family health, as an important part of individual health, may play a mediating role between WFC and mental health (Arik Tasyikan and Demiral, 2022). Family health includes not only the physical health of family members but also their mental health and the harmony of family relationships (Killien, 2004). Research has shown that good family health can provide emotional support for individuals, thereby helping to reduce the negative impact of work-life conflict (Sobol and Ben-Shlomo, 2019). For example, a harmonious family environment can provide a place for working people to relax and recover, and the support and understanding of family members can help them better cope with work pressure (Chen et al., 2022). Family support mechanisms, which include emotional and practical forms of assistance, play a crucial role in helping employees manage WFC (Al-Hammouri and Rababah, 2024). These mechanisms, encompassing spousal support, grandparental care, shared responsibilities, and mutual understanding among family members (Nasharudin and Rui, 2024), represent a particularly promising yet underexplored candidate mechanism. It remains unclear whether WFC erodes family health, which in turn elevates depression risk, or whether family health is merely correlated with this association. Elucidating this mechanism is critical because, if family health operates as a mediator, it constitutes a concrete, modifiable target for family-centered mental health interventions. These unresolved issues suggest three specific gaps in the current literature that can be further explored.

### Research gaps

Three specific gaps in the current literature motivate this investigation.

First, a mechanistic gap. While numerous studies have identified social support, psychological capital, or coping strategies as parallel correlates of the WFC–depression association, the mediating role of family health has rarely been subjected to rigorous empirical scrutiny.

Second, a theoretical gap. Although some theoretical models provide compelling frameworks for the mediation effect, these perspectives have not been systematically integrated to explain how family health functions as a resource caravan that channels WFC into mental health outcomes.

Third, a contextual gap. The existing evidence is overwhelmingly derived from small-scale, occupation-specific studies (e.g., nurses or teachers). Large-scale, nationally representative investigations remain scarce.

### Research objectives and questions

Guided by the aforementioned gaps, this study aims to investigate the mediating role of family health in the relationship between WFC and depression among Chinese working parents. It seeks to answer the following research questions:

Question 1: Is WFC significantly associated with depression among Chinese working parents after controlling for demographic and health-related confounders?

Question 2: Is WFC negatively associated with family health, and does family health predict lower depression?

Question 3: Does family health partially mediate the relationship between WFC and depression, and what proportion of the total effect is explained by this indirect pathway?

### Study contributions

This study offers three contributions to the literature. Theoretically, it advances an integrated resource-based framework by positioning family health as a dynamic mediating resource that channels WFC into depressive symptomatology. Empirically, it provides a large-scale, nationally representative estimate of the indirect effect of WFC on depression via family health in a Chinese working-parent sample, addressing the small-sample limitations that dominate existing research. Practically, by quantifying the precise proportion of the WFC–depression association attributable to family health, the study identifies an intervention target and offers actionable metrics for occupational mental health screening and public health policy.

## Literature review

### Work–family conflict and depression: empirical evidence

The association between WFC and adverse mental health outcomes has been among the most documented findings in occupational health psychology. A substantial body of cross-sectional and longitudinal research has established that employees who experience greater difficulty balancing work and family life report elevated levels of psychological distress, emotional exhaustion, and depressive symptomatology. A national survey in the USA showed that employees who reported experiencing WFC often were 1.99–29.66 times more likely than were employees who reported no WFC to experience a clinically significant mental health problem (Frone, 2000). Longitudinal studies also revealed that mental health trajectories closely track changes in WFC over time. Parents experiencing chronic WFC across multiple time points reported the poorest mental health outcomes, while those who experienced relief from conflict showed significant improvements in mental health (Cooklin et al., 2016). Individual differences in reactivity to daily WFC were associated with psychological disorders, independent of baseline negative affect. This suggests that not just the occurrence of conflict, but how individuals process and respond to it emotionally, shapes mental health outcomes (Lawson et al., 2021).

### Family health as a protective resource

Family health represents a relatively recent but increasingly influential construct in public health. Empirical research has consistently demonstrated that higher levels of family health are associated with superior individual health outcomes. A harmonious family environment can provide emotional refuge and practical assistance, enabling working individuals to recover from work-related stress and replenish depleted psychological resources. Family support mechanisms—including spousal support, grandparental care, and shared domestic responsibilities—play a crucial role in helping employees manage WFC (Chen et al., 2022). However, despite these advances, family health has rarely been positioned as a mediating mechanism in the relationship between WFC and mental health. Most studies have treated it either as an outcome to be predicted or as a moderator that buffers or exacerbates existing associations. The question of whether family health actively transmits the effect of WFC on depression remains unresolved.

### Theoretical framework

Guided by the Conservation of Resources (COR) theory (Hobfoll, 1989) and the Work–Home Resources (W-HR) model (Ten Brummelhuis and Bakker, 2012), we posit that family health functions as a key “resource caravan”: when work demands deplete psychological energy, the deterioration of family health transmits this resource loss into depressive symptomatology. However, this theoretical proposition has yet to be empirically quantified in a large-scale, nationally representative sample of working parents. COR theory states that individuals strive to obtain, retain, and protect resources. This resource gain can offset loss effects toward depression. WFC constitutes a significant resource drain. When work demands encroach upon family time and energy, individuals lose the opportunity to recover, to engage in restorative family activities, and to benefit from emotional support. Family health can replenish psychological energy depleted by work demands. Likewise, according to the W-HR model, abundant resources can directly enhance personal resources and indirectly buffer the impact of WFC on mental health. Integrating these perspectives, we hypothesize that family health can be regarded as a key resource that buffers the negative impact of resource loss in the work domain on mental health. When WFC depletes this resource through the mechanisms described above, the buffering capacity of the home environment is compromised, and the individual’s personal resources (e.g., emotional regulation capacity, sense of mastery) are diminished. This depletion renders the individual more susceptible to depression. Family health not only matters for mental health outcomes but also serves as a transmission belt that carries the negative effects of WFC into individual psychopathology.

### Hypotheses development

Drawing upon the evidence reviewed above and the integrated theoretical framework, we derive three testable hypotheses:

*H1*: WFC is positively associated with depression among working parents. Higher levels of WFC predict increased risk of depression after controlling for demographic and related confounders.

*H2*: WFC is negatively associated with family health. Higher levels of WFC predict lower family health scores.

*H3*: Family health partially mediates the relationship between WFC and depression, such that (a) WFC negatively predicts family health, and (b) family health negatively predicts depression, with the indirect effect accounting for a significant proportion of the total effect.

Therefore, this study aims to extend our understanding of the relationship between WFC and depression among working parents, with a particular focus on the mediating role of family health. The findings of this study will provide a new perspective for understanding the mental health problems of working individuals and offer scientific measures for developing effective mental health interventions.

## Methods

### Ethical considerations

Ethical approval for this study was obtained from the Institutional Review Board (IRB) of the Health Culture Research Center, a key research base of philosophy and social sciences at Shaanxi University (Approval No. JKWH-2021-01; Date of approval: March 1, 2021). The study commenced only after ethical approval was granted. The study was conducted in accordance with the Declaration of Helsinki of the World Medical Association.

The questionnaire was an anonymous survey and did not collect any identity information, such as the respondent’s name, phone number, address, or ID number, to ensure complete anonymity. Informed consent was obtained electronically and orally from every participant before any data were recorded. The study used a multi-step consent procedure embedded in the data collection platform. Prior to data collection, members of the research team clearly explained to each participant, in a face-to-face manner, the objectives of the research, the confidential and anonymous nature of data handling, the intended use of the findings for academic publication, and the fact that participation involved no foreseeable physical or psychological risks. After clicking the survey link, respondents first viewed a dedicated consent page that contained information covering the study’s purpose, data use, potential risks, confidentiality, scope of consent, plans for future academic publication, and the right to withdraw at any time without penalty. Participants were required to answer the question “Do you agree to participate in this survey?” Only participants who selected “Yes” were allowed to proceed. The system automatically time-stamped the consent choice and generated a unique questionnaire ID that was linked to the subsequent answers. Those who chose “No” were directed to a blank submission. No uniform compensation scheme was specified in advance. Participation remained entirely voluntary and could be terminated at any time without penalty.

### Participants

The data for our study were extracted from the 2021 Psychology and Behavior Investigation of Chinese Residents (PBICR) (Wu et al., 2024). The survey project aimed to establish a database through a multicenter, large-sample, cross-sectional survey to provide robust data for research in various fields and for a comprehensive and systematic understanding of the public’s health—both physical and mental. The data used in our study were collected in 2021, and we obtained permission to use them for analysis on August 11, 2024, after submitting an application through the project website.

The investigation was conducted in 23 provinces, 5 autonomous regions, and 4 municipalities directly under the central government from July 10, 2021 to September 15, 2021. Based on the data results of “the seventh national population census in 2020,” quota sampling (with quota attributes of gender, age, and urban–rural distribution) was conducted across 120 cities, so that the gender, age and urban–rural distribution of the samples basically conformed to the national characteristics. The sampled cities were stratified to cover the eastern, central, and western economic zones, ensuring representation of regions with high, medium, and low GDP (Gross Domestic Product) per capita. The first stage involved equal probability sampling, in which four municipalities (Beijing, Tianjin, Shanghai, and Chongqing) were directly included in the study. For the 22 provinces and 5 autonomous regions, a sampling frame was used to determine the number of cities to be sampled based on each province’s or autonomous region’s population size, and 2 to 12 cities were randomly selected using a random number table method. A total of 120 cities were sampled in the first stage. In the second stage, probability sampling was used to determine the number of communities to be sampled within the 120 selected cities, with an urban-to-rural ratio of 3:2. The third stage involved quota sampling of the population within each selected community. The quota attributes were gender and age, with a gender ratio of 1:1 and an age distribution that closely aligns with China’s “population pyramid.”

Regarding the sampling justification, a multistage hybrid sampling framework was employed to balance statistical representativeness with fieldwork feasibility across China’s vast administrative divisions. Probability sampling at the provincial and municipal stages ensured broad geographical coverage and randomization, while quota sampling at the individual level (aligned with the Seventh National Population Census) corrects for demographic skewness in sex, age, and urban–rural distribution (Wu et al., 2024). This combined approach is widely adopted in large-scale mental health and behavioral surveys in China and has been detailed in the PBICR methodological protocol (Yi et al., 2025).

For the recruitment and field survey, the investigators first contacted the selected community health centers or neighborhood committees to establish local survey sites. Recruitment was carried out by posting paper flyers and circulating electronic notices. Second, investigators verified the identities of potential participants, ensuring they met the inclusion criteria and were not excluded by any criteria. Third, each qualified individual completed the electronic questionnaire in a face-to-face, one-to-one session. The questionnaire was accessed by scanning a QR code or clicking a link. Informed consent was obtained at the beginning of the survey. The questionnaire number was provided by the investigators. If a participant was cognitively capable but unable to complete the form independently, the investigator conducted an individual interview and recorded the responses on the participant’s behalf. For data collection, each city engaged at least one surveyor or a group of surveyors tasked with distributing and collecting questionnaires. Individual surveyors were responsible for collecting 30 to 90 questionnaires, while groups aimed to collect 100–200. The surveyors used the Online Questionnaire Star platform^1^ to distribute the questionnaire to each participant, obtain informed consent from each respondent, and record the questionnaire number issued to that individual. A total of 11,031 valid questionnaire data were obtained during the investigation period (from July 10, 2021 to September 15, 2021) that had high quality and accurate national representation (He et al., 2022).

The inclusion criteria for the sample in this study were as follows: (1) being aged 18 years or older; (2) having nationality of the People’s Republic of China; (3) residency in China (with an annual absence from home not exceeding 1 month); (4) being part of the working population, including individuals employed full-time or without fixed employment; (5) having one or more children. The exclusion criteria were as follows: (1) students; (2) retirees; (3) individuals without children; (4) individuals unwilling to participate; (5) individuals missing key variables, such as WFC, family health, PHQ-9 score.

These criteria were established to ensure that all participants possessed the prerequisite role configurations for experiencing WFC and that the sample remained culturally and demographically coherent. Age ≥18 years was required for informed consent and stable occupational and family roles. Chinese nationality and permanent residency were stipulated to maintain cultural homogeneity, because WFC and family health are shaped by context-specific factors such as multigenerational interdependence and household registration systems. Employment status (including non-standard employment) was essential because the study’s central construct, WFC, presupposed the simultaneous holding of work and family roles; excluding informal workers would introduce selection bias against vulnerable labor groups. The presence of children was required because the FHS-SF was operationalized within a family-system context (Lin et al., 2025). Students and retirees were excluded because they did not concurrently hold active work and parental roles, rendering WFC theoretically irrelevant to them. Finally, cases with missing data on key analytical variables were excluded to preserve statistical power and prevent bias from unknown missing mechanisms.

**Figure 1:**
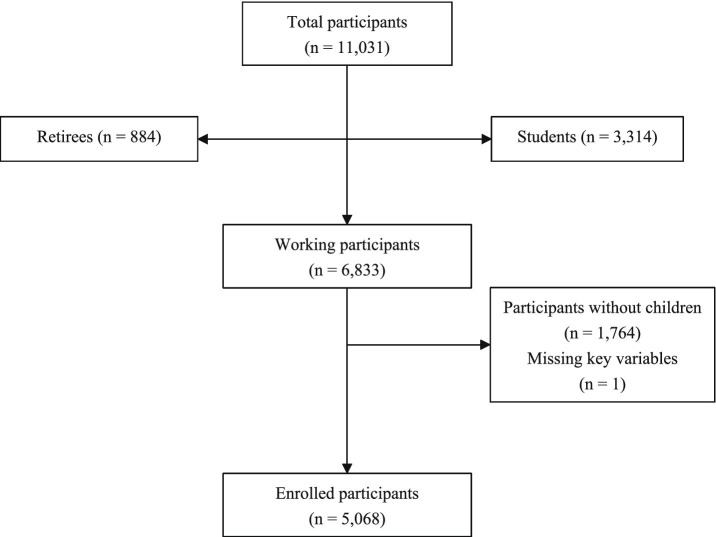
Flow chart of participant selection for the cross-sectional study examining work–family conflict, family health, and depression among Chinese working parents in 120 cities.

Based on the sample inclusion and exclusion criteria, research data from 5,068 working parents were ultimately included in this study. See Figure 1.

### Measures

The Patient Health Questionnaire-9 (PHQ-9), a 9-item instrument developed by Kroenke et al. (2001) was used to evaluate depressive symptoms in the study. The PHQ-9 is widely used as a screening tool for assessing the severity of depression in both clinical and research settings. The scale has gained widespread use in clinical settings and various studies in China (Yang et al., 2025). The PHQ-9 is a 4-point rating scale, with response options ranging from “not at all (= 0)” to “nearly every day (= 3).” The scores on the PHQ-9 ranged between 0 and 27. The standard cut-off score for screening for possible major depression was 10 or above in this study. Cronbach’s *α* of PHQ-9 in this study was 0.930.

The Work–Family Conflict Scale (WAFCS) (Haslam et al., 2015) contains two dimensions: work-to-family conflict and family-to-work conflict. Five items for each dimension were rated on a 7-point scale (1 = very strongly disagree, 7 = very strongly agree). Each dimension was scored according to the sum of its items, and the total score was determined based on the sum of the points from each dimension, with higher scores indicating higher levels of WFC. The bidirectional nature of WFC, in which separate dimensions are highly correlated, means they may exert synergistic effects on mental health. In the present study, the two subscales were strongly correlated (*r* = 0.752, *p* < 0.001). We operationalized WFC as a composite construct reflecting the overall burden of bidirectional role conflict, consistent with the recommended approach and validated in subsequent Chinese-language applications. The scale has been translated into Chinese and used in different fields (Li et al., 2025). Cronbach’s *α* in this study was 0.945.

Family health was measured using the Short Form of the Family Health Scale (FHS-SF) (Crandall et al., 2020). The Chinese version of FHS-SF has good reliability and validity and can be used to assess the level of family health among Chinese residents (Chang et al., 2024; Wang et al., 2022). Wang et al. (2022) reported the Cronbach’s *α* for the FHS-SF as 0.83 in the same investigation program. Additionally, the Cronbach’s α for the four subscales ranged from 0.70 to 0.90. The standardized factor loadings in the confirmatory factor analysis were all above 0.50, with GFI = 0.98; NFI = 0.97; RFI = 0.95; RMSEA = 0.07. It contains four dimensions: (1) family, social, or emotional health processes; (2) family healthy lifestyle; (3) family health resources; and (4) family external social support. The calculated score ranged from 10 to 50, with a higher score indicating better family health. In our study, the Cronbach’s *α* for the FHS-SF was 0.847. The reliability values for the 4 dimensions were 0.914, 0.882, 0.756, and 0.741, respectively, indicating acceptable reliability. This study used the total score of the scale for the mediation analysis.

The 7-item Generalized Anxiety Disorder Scale (GAD-7), developed by Spitzer (Spitzer et al., 2006), has gained widespread use in clinical settings and various studies in China (Huang et al., 2023), particularly for screening anxiety disorders. The GAD-7 comprises seven items that assess symptoms of generalized anxiety, such as “feeling tense, anxious, or on edge” and “not being able to stop or control worrying.” Participants were asked how often they had experienced these symptoms over the past 2 weeks, according to their experience. The scale was rated on a four-point scale, with responses ranging from “0 = not at all” to “3 = almost every day” (Cronbach’s α = 0.946). Higher scores on the scale indicate a higher level of anxiety severity.

### Covariates

To assess the influence of potential confounding factors, we selected several important covariates–including sex, age, education, marital status, occupational status, family structure, family income, number of children, number of illnesses and medical insurance–that were collected in the questionnaires.

In this study, Han ethnicity accounted for the vast majority, with different ethnic minorities comprising 0–1.1%. There was no statistically significant difference in the depression rate between the Han majority and ethnic minorities (χ^2^ = 1.581, *p* = 0.209). Therefore, we did not include ethnicity as a covariate.

### Statistical analysis

The gender, age, and urban–rural distribution of the samples basically conformed to the demographic characteristics of China. Participants were divided into 2 groups based on the level of depression. Depression was operationalized as a binary variable using the standard PHQ-9 cutoff score of ≥10. Baseline variable differences were tested by chi-square tests. The relationship of depression with WFC and family health was explored using logistic regression models in this study. Model 1 was adjusted for sex; Model 2 was further adjusted for age group and marital status building on Model 1. Model 3 added family structure, number of illnesses, occupational status, and anxiety to Model 2. Odds ratios (ORs) and 95% CI for the risk of depression were estimated. Collinearity diagnostics indicated no multicollinearity concerns (VIF range: 1.04–2.27). The PROCESS Macro (Model 4) with bootstrapping was employed to estimate direct and indirect effects and to investigate the mediating role of family health. A total of 5,000 bootstrap samples were used in this study. The significance level was set at *p* < 0.05 for all hypothesis tests. Data were analyzed using SPSS 22.0 (IBM Corp., Armonk, NY, United States) and PROCESS Macro Version 4.1 (Model 4) (Bonacquisti et al., 2024).

## Results

### Participant demographics

Among the 5,068 surveyed participants, 4,258 reported no depression, accounting for 84.0%, while 810 (16.0%) reported depression. The results indicated that depression has gradually become a common mental health issue among the working population. Table 1 shows the differences in depression distribution across groups with different demographic characteristics. Among different age groups of the surveyed population, the group aged under 35 had a significantly higher proportion of depression (20.43%) than other age groups (12.28–17.46%). When WFC increased, individuals were more likely to experience depression. Among different marital status groups, the proportion of singles (including never-married, divorced, and widowed) facing depression was significantly higher than that of married individuals (or those living with a partner). The rate of depression in the single group was 25.08%, compared to 15.36% in the married group. A lower proportion of depression was reported by 13.61% of participants from core families compared to those from other family structures, with statistically significant differences (*p* < 0.001). Core families consist of a married couple and their unmarried children, representing the traditional family structure in China. Family structure and marital status may influence how individuals experience WFC. As the number of illnesses increased, the proportion of parents experiencing depression also gradually increased. Among parents with 2 or more illnesses, 27.34% experienced depression. Compared with the “no depression” group, participants in the depression group had significantly higher scores of WFC and anxiety (GAD-7), and lower scores of family health (FHS-SF) (*p* < 0.001). There was no statistically significant difference in the depression rates among those with different levels of family income, gender, education, number of children, or occupational status (*p* > 0.05). See Table 1.

**Table 1:** Baseline characteristics of 5,068 working parents in a cross-sectional survey across 120 Chinese cities, 2021.

Variables	Total (*n* = 5,068)	No Depression (*n* = 4,258)	Depression (*n* = 810)	Statistic	*p*
Sex, *n* (%)				χ^2^(1) = 3.28	0.070
Female	2,719 (53.65)	2,308 (84.88)	411 (15.12)	Phi = 0.025	
Male	2,349 (46.35)	1950 (83.01)	399 (16.99)		
Age group (year), *n* (%)				χ^2^(3) = 33.43	**<0.001**
<35	876 (17.28)	697 (79.57)	179 (20.43)	V = 0.081	
35~	1838 (36.27)	1,517 (82.54)	321 (17.46)		
45~	1702 (33.58)	1,493 (87.72)	209 (12.28)		
55~	652 (12.87)	551 (84.51)	101 (15.49)		
Education, *n* (%)				χ^2^(3) = 4.67	0.197
Junior or less	1,676 (33.07)	1,429 (85.26)	247 (14.74)	V = 0.030	
High school	1,037 (20.46)	864 (83.32)	173 (16.68)		
Junior college	823 (16.24)	698 (84.81)	125 (15.19)		
Bachelor or more	1,532 (30.23)	1,267 (82.70)	265 (17.30)		
Marital status, *n* (%)				χ^2^(1) = 21.25	**<0.001**
Single/divorced/widow	323 (6.37)	242 (74.92)	81 (25.08)	Phi = 0.065	
Married	4,745 (93.63)	4,016 (84.64)	729 (15.36)		
Number of children, *n* (%)				χ^2^(2) = 2.25	0.324
1	2,696 (53.20)	2,279 (84.53)	417 (15.47)	V = 0.021	
2	1910 (37.69)	1,601 (83.82)	309 (16.18)		
3 or more	462 (9.12)	378 (81.82)	84 (18.18)		
Occupational status, *n* (%)				χ^2^(1) = 3.77	0.052
Employed full time	3,286 (64.84)	2,785 (84.75)	501 (15.25)	Phi = 0.027	
No fixed employment	1782 (35.16)	1,473 (82.66)	309 (17.34)		
Number of illnesses, n (%)				χ^2^(2) = 45.02	**<0.001**
0	3,802 (75.02)	3,250 (85.48)	552 (14.52)	V = 0.094	
1	871 (17.19)	721 (82.78)	150 (17.22)		
2 or more	395 (7.79)	287 (72.66)	108 (27.34)		
Family type, *n* (%)				χ^2^(1) = 33.39	**<0.001**
Other family type	1968 (38.83)	1,580 (80.28)	388 (19.72)	Phi = 0.081	
Core family	3,100 (61.17)	2,678 (86.39)	422 (13.61)		
Family income, *n* (%)				χ^2^(3) = 3.90	0.272
<3,000	1,492 (29.44)	1,235 (29.00)	257 (31.73)	*V* = 0.028	
3,000~	2007 (39.60)	1705 (40.04)	302 (37.28)		
6,000~	1,241 (24.49)	1,048 (24.61)	193 (23.83)		
12,000~	328 (6.47)	270 (6.34)	58 (7.16)		
WFC, Mean ± SD	33.47 ± 12.85	31.74 ± 12.12	42.54 ± 12.75	*t*(5066) = −23.05 Cohen’s *d* = −0.88	<0.001
FHS-SF, Mean ± SD	38.36 ± 6.53	39.15 ± 6.41	34.20 ± 5.47	*t*(5066) = 22.94 Cohen’s *d* = 0.79	<0.001
GAD-7, Mean ± SD	4.12 ± 4.29	2.90 ± 2.98	10.50 ± 4.45	*t*(5066) = −46.71 Cohen’s *d* = −2.33	<0.001

*t*: *t*-test, χ^2^: Chi-square test, SD: standard deviation.

### The impact of work–family conflict and family health on depression

In this study, we explored logistic regression models to analyze the impact of WFC and family health on depression. The results in Model 1 (sex as a covariate) showed that both WFC and family health significantly affected depression (*p* < 0.001), but in opposite directions. Higher WFC scores were significantly associated with an increased risk of depression (OR = 1.08, 95% CI: 1.07–1.09). Conversely, higher family health scores were significantly associated with a reduced risk of depression (OR = 0.88, 95% CI: 0.87–0.89). With more covariates (age group and marital status) entered in Model 2, the impact of WFC and family health on depression remained significant (OR = 1.07, 95% CI: 1.06–1.08, and OR = 0.90, 95% CI: 0.88–0.91). We further included more potential influencing factors (family structure, number of illnesses, occupational status, and anxiety) in Model 3. The results showed the OR of WFC decreased slightly but remained significant (OR = 1.01, 95% CI: 1.01–1.02). Higher levels of WFC increased the risk of depression. The OR of FHS-SF increased to 0.95 (95% CI: 0.93–0.97, *p* < 0.001), maintaining the same direction of impact as in Model 1. In this model, age, number of illnesses, and anxiety were also significantly associated with depression. The risk of depression decreased significantly in age groups over 40 (OR = 0.61, 95% CI: 0.44–0.85) and over 50 (OR = 0.57, 95% CI: 0.36–0.89). Participants with 2 or more illnesses had a significantly higher risk of depression (OR = 2.40, 95% CI: 1.67–3.47). In addition, anxiety was also associated with depression. Higher GAD-7 scores significantly increased the risk of depression (OR = 1.71, 95% CI: 1.64–1.79). See Table 2.

**Table 2:** Association between WFC, family health and depression among working parents in a cross-sectional survey (*n* = 5,068) in the Logistic model.

Predictor variables	Model 1		Model 2		Model 3	
	OR (95% CI)	*p*-value	OR (95% CI)	*p*-value	OR (95% CI)	*p*-value
WFC	1.07 (1.06–1.08)	<0.001	1.07 (1.06–1.08)	<0.001	1.01 (1.01–1.02)	0.038
FHS-SF	0.89 (0.88–0.91)	<0.001	0.90 (0.88–0.91)	<0.001	0.95 (0.93–0.97)	<0.001
Sex (ref: female)						
Male	0.96 (0.82–1.13)	0.064	1.01 (0.85–1.19)	0.944	1.21 (0.97–1.52)	0.089
Age (ref: <35 year)						
35-	N/A	N/A	0.91 (0.72–1.13)	0.391	0.85 (0.63–1.15)	0.299
45-	N/A	N/A	0.68 (0.54–0.87)	0.002	0.61 (0.44–0.85)	0.004
55-	N/A	N/A	1.02 (0.76–1.38)	0.882	0.57 (0.36–0.89)	0.013
Marital status (ref: single/divorced/widow)						
Married	N/A	N/A	0.61 (0.45–0.82)	0.001	0.91 (0.60–1.39)	0.672
Family type (ref: other family type)						
Core family	N/A	N/A	N/A	N/A	0.94 (0.74–1.19)	0.621
Number of illnesses (ref: 0)						
1	N/A	N/A	N/A	N/A	1.12 (0.84–1.51)	0.438
2 or more	N/A	N/A	N/A	N/A	2.40 (1.67–3.47)	<0.001
Occupational status (ref: Employed full time)						
No fixed employment	N/A	N/A	N/A	N/A	0.89 (0.70–1.14)	0.356
GAD-7	N/A	N/A	N/A	N/A	1.71 (1.64–1.79)	<0.001

OR: Odds Ratio, CI: Confidence Interval.

### The correlation between work–family conflict, family health, and depression scores

The bivariate correlation analysis results showed that there was a statistically significant correlation between WFC, family health, and depression (*p* < 0.001). WFC was positively correlated with depression (*r* = 0.443), and family health was negatively correlated with depression (*r* = −0.335).

### The mediating effect of family health in the relationship between work–family conflict and depression

**Figure 2:**
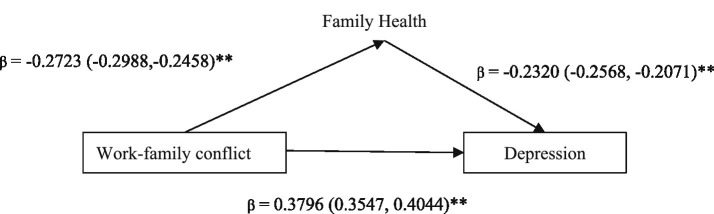
Standardized path coefficients for the mediation model examining family health as a mediator between work–family conflict (WFC) and depression among Chinese working parents (*n* = 5,068). All variables were standardized as *Z*-scores prior to analysis. Values on paths represent standardized regression coefficients (*β*). The indirect effect of WFC on depression via family health was *β* = 0.0632, accounting for 14.27% of the total effect. Bootstrap 95% CI for indirect effect: 0.0541–0.0734. *p* < 0.01.

Model 4 (a simple mediation model) in the SPSS macro developed by Hayes was used to test the mediating effect of family health in the relationship between WFC and depression. All variables were standardized as *Z*-scores before conducting the mediation model test. The results were shown in Table 3 and Table 4. The negative effect of WFC on family health was significant (*β* = −0.2723, *t* = −20.1394, *p* < 0.01). The negative predictive effect of family health on depression was also significant (*β* = −0.2320, *t* = −18.3018, *p* < 0.01). The positive predictive effect of WFC on depression was significant (*β* = 0.4428, *t* = 31.1595, *p* < 0.01). After including the mediator, the direct effect of WFC on depression was 0.3796, and the total effect was 0.4428. The direct effect accounted for 85.73% of the total effect, while the indirect effect accounted for 14.27%. In addition, the upper and lower limits of the 95% bootstrap confidence interval for the mediating effect of family health did not include 0 (see Table 4), indicating that a significant partial mediation effect existed. The results indicated that family health partially mediates the relationship between WFC and depression, meaning that WFC not only indirectly affects depression through family health but also has a direct positive effect on depression. The model explained 24.60% of the variance in depression (*R*^2^ = 0.2460), and the overall model fit was significant (*F* = 826.319, *p* < 0.001). The mediation model is shown in Figure 2.

**Table 3:** Mediation model of family health in the association between work–family conflict and depression among working parents, 2021.

Constant	Depression			Family health			Depression		
	*β*	se	*t*	*β*	se	*t*	*β*	se	*t*
WFC	0.4428**	0.0122	31.1595	– 0.2723**	0.0135	−20.1394	0.3796**	0.0127	29.9456
Family health							−0.2320**	0.0127	−18.3018
*R* ^2^	0.1962			0.074			0.2460		
*F*(df1, df2) value	*F*(1, 5,066) = 1236.190**			*F*(1, 5,066) = 405.596**			*F*(2, 5,065) = 826.319**		

**p < 0.01.

**Table 4:** Decomposition of total, mediated and direct effects of the mediating role of family health in the relationship between WFC and depression among working parents.

Effect	Path way	Standardized Effect value	Effect proportion (%)	95%CI		*p*-value
				Lower	Upper	
Total effect		0.4428	100.00	0.411	0.4674	<0.001
Direct effect	WFC → Depression	0.3796	85.73	0.3547	0.4044	<0.001
Indirect effect	WFC → Family Health→Depression	0.0632	14.27	0.0541	0.0734	<0.05

## Discussion

Based on previous research, we verified that increased WFC is associated with more severe depressive symptoms and further expanded the scope of existing research. This study advances the literature by identifying family health as a measurable transmission mechanism through which WFC translates into depression. The impact of WFC on depression extends to both immediate and long-term effects on mental health, affecting not only individual well-being but also organizational outcomes (Zhang et al., 2023). This relationship is particularly concerning given that depression among working adults can lead to decreased productivity, increased absenteeism, and potential career stagnation (Van Gordon et al., 2017). In our study, a notable proportion of both sexes experienced depression, highlighting the widespread nature of this issue. The findings suggest that role overload and time commitments are key contributors to WFC. The demands of managing multiple overlapping roles create significant psychological tension, making it difficult for employees to balance work and family responsibilities (Mark et al., 2024). This is further exacerbated by the lack of support systems, both at work and within the family, which would otherwise act as facilitators in work-family relationships. Lack of supervisor, co-worker, and family support can significantly increase stress associated with WFC. These absent support systems may serve as stressors rather than facilitators (Noroozi et al., 2023). In our study Family health emerged as a significant protective factor against depression. Its mediating role clarifies how the internal operation of a family can transform WFC into a risk for depression. Family health encompasses not only the physical well-being of family members but also the emotional and relational aspects of family life (Ramaswami et al., 2022). The mediating effect of family health further highlights its mechanistic importance in the WFC–depression relationship. This finding underscores the need for holistic interventions that simultaneously address individual stress management and family-level resource replenishment, rather than treating employees in isolation from their families.

The observed 14.27% mediation is modest but practically meaningful. In the context of modern society, rapid urbanization compresses extended-family support into a single household, making family health a critical and consequential resource. The remaining 85.73% direct effect underscores that WFC also affects depression through neuroendocrine stress pathways, sleep disruption, or workplace burnout that bypass family dynamics. Consequently, integrated interventions should simultaneously safeguard family boundaries (to replenish the 14% resource buffer) and address issues in job design and enhance supervisor support. The findings corroborate COR theory and the W-HR model. Resource loss in one life domain (work-to-family conflict) depletes individuals’ resource caravans, thereby increasing their vulnerability to psychological distress. The significant indirect pathway that we quantified illustrates this loss spiral. Likewise, the W-HR model predicts that family health can counteract the negative impact of work demands on mental health; the mediation effect we observed empirically supports this buffering proposition.

Our study also revealed that younger parents (under 35 years old) had a significantly higher rate of depression (20.43%) compared to other age groups. This finding suggests that younger individuals, especially those with young children, face unique challenges in balancing work and family life (Aviv et al., 2025). Younger parents often need to invest more time and energy in caring for their children, which can exacerbate WFC and lead to higher levels of depression. Additionally, younger employees may have less experience in managing work-life balance and may lack the necessary support systems, both at work and at home, to cope with these challenges (Rinne et al., 2024). However, the number of children did not predict depression. Among married parents, additional children may have their burden offset by co-parenting resources, whereas among single parents, even one child represents an unshared burden. These patterns suggest that age and marital status operate through different mechanisms: the former through life-course stage and resource accumulation, the latter through ruptures in social support network.

A significant association between the number of illnesses and the risk of depression among working parents was also observed in this study. These additional stressors and time commitments can aggravate WFC and contribute to higher levels of depression. This finding underscores the need for workplace policies and support systems that recognize and address the challenges faced by employees with illnesses.

The association between anxiety and depression served as a statistical control strategy: even after accounting for this well-established comorbidity, WFC and family health retained independent effects on depression. This pattern suggests that anxiety and depression often co-occur and share common underlying mechanisms (Wong et al., 2024). High levels of anxiety associated with WFC can lead to both anxiety and depression, creating a vicious cycle where one condition aggravates the other. Interventions aimed at improving family health and reducing WFC may positively impact both anxiety and depression. These findings converge on a common policy implication: mental health interventions must operate at both individual and family levels to be effective.

The findings of this study have significant implications for both organizational practices and public policy. At the organizational level, our results suggest that targeted interventions aimed at reducing WFC can have a positive impact on employee mental health. Supervisor training programs that promote family-supportive behaviors have been shown to be particularly effective in reducing WFC (Bowman et al., 2024). Additionally, flexible scheduling and family-friendly policies can help employees better manage their work and family responsibilities, thereby reducing conflict and its negative outcomes (Sasaki et al., 2025). From a policy perspective, our study highlights the importance of comprehensive support systems that address both individual- and family-level needs. This includes personal-level resources such as anxiety management and flexible schedules, as well as organizational-level support through family-friendly policies (Khajeh and Nakhaee, 2024). For specific workforce segments, such as young parents and frontline workers, targeted support measures like family caregiving assistance and financial subsidies can yield significant benefits (DePasquale et al., 2018). These interventions not only reduce WFC but also improve work engagement and employee health, ultimately benefiting both individuals and organizations.

### Limitations and future directions

Several key covariates were not measured in this study could confound the observed associations. Work-side factors such as supervisor support, flexible scheduling, and utilization of parental leave could simultaneously reduce WFC and depressive symptoms, thereby potentially inflating the crude effect of WFC on depression. Similarly, family-side stressors such as a spouse’s long working hours, the absence of grandparental childcare, or objective measures of monthly household income may erode family health and heighten depression risk, creating spurious correlations. At the individual level, pre-existing mental disorders or low medication adherence could precede both heightened WFC reports and current depression, producing apparent confounding. Because these variables were not collected in the 2021 PBICR survey, our estimates might reflect, in part, uncontrolled confounding rather than the true causal effect of WFC. Future investigations should incorporate more key variables. Including such key covariates is likely to offer a more precise assessment of the mediation pathway via family health. Limitations in causal inference should be explicitly acknowledged in this study. The cross-sectional design captures WFC, family health, and depression concurrently, thereby precluding the determination of temporal ordering, which is a prerequisite for establishing a mediation effect (Maxwell and Cole, 2007). Consequently, the indirect effect of family health should be interpreted as a statistical decomposition of concurrent associations rather than as evidence of a causal indirect pathway. It remains plausible that (a) WFC erodes family health and subsequently precipitates depression, (b) pre-existing depressive symptoms impair family functioning and thus intensify perceived WFC, or (c) a bidirectional process exists. Additionally, unmeasured time-varying confounders (e.g., sudden job loss, childcare disruptions, or other life events) may simultaneously influence WFC, family health, and depressive symptomatology. Future longitudinal or cohort studies with multiple measurement waves are required both to determine the direction of influence and to establish whether family health functions as a true mediator, a proxy marker, or a consequence of the WFC–depression relationship. While WFC conceptually captures two dimensions, longitudinal research should resolve these dimensions to test whether work-to-family conflict erodes family health more severely than family-to-work conflict, or whether family health buffers one direction more effectively than the other. Additionally, our study focused on working parents in China, which limits the generalizability of the findings to other populations. Further research is needed to explore the role of cultural and contextual factors in shaping the relationship between WFC and mental health outcomes.

In conclusion, our study provides valuable insights into the complex relationships among work–family conflict, family health, and depression. Family health accounts for 14.27% of the total effect, which holds public health significance given the large sample size and the robustness of the effect. The findings emphasize the importance of both individual- and family-level support in mitigating the negative impact of WFC on mental health. By addressing these factors through targeted interventions and policies, we may create a more supportive environment that promotes the well-being of working individuals and their families.

## Data Availability

The raw data supporting the conclusions of this article will be made available by the authors, without undue reservation.

## Footnotes

1
https://www.wjx.cn/
